# Relationship between serum estrogen levels and blood stasis syndrome in postmenopausal women with coronary heart disease

**DOI:** 10.12669/pjms.311.5490

**Published:** 2015

**Authors:** Xin Liu, Chunyu Guo, Xiaojuan Ma, Rui Tian, Ying Zhang, Huijun Yin

**Affiliations:** 1 Xin Liu, China Academy of Chinese Medical Sciences, Beijing (100700), China.; 2 Chunyu Guo, Center of Cardiovascular Diseases, Xiyuan Hospital,Beijing (100091), China.; 3 Xiaojuan Ma, Center of Cardiovascular Diseases, Xiyuan Hospital,Beijing (100091), China.; 4 Rui Tian, Beijing Anzhen Hospital, Beijing (100029), China.; 5 Ying Zhang, China Academy of Chinese Medical Sciences, Beijing (100700), China.; 6 Huijun Yin, China Academy of Chinese Medical Sciences, Beijing (100700), China.

**Keywords:** Coronary Heart Disease, Blood stasis syndrome, Postmenopausal woman, estrogen, ICAM-1

## Abstract

**Objectives:**

To investigate the difference of serum estrogen, serum lipids and inflammatory factors levels in postmenopausal women with coronary heart blood stasis syndrome and non-blood stasis syndrome.

**Methods:**

Twenty five healthy postmenopausal women were selected as a healthy control group who were compared with 43 postmenopausal women with coronary heart disease (CHD) first visiting a doctor for the CHD. Among the postmenopausal women with CHD, There were 23 patients with blood stasis syndrome (BSS) and 20 patients with non-blood stasis syndrome (NBSS). The levels of plasma triglyceride (TG), total cholesterol (TC) were determined in blood samples taken after patients’ admission in Beijing Anzhen Hospital. The serum estradiol(E_2_) was measured by electrochemiluminescence assay and soluble intercellular adhesion molecule-1(sICAM-1) was measured by enzyme-linked immune sorbent assay (ELISA).

**Results:**

Compared with the healthy control group, the levels of TG and TC, sICAM-1 in coronary heart disease group were all significantly increased (*P*<0.05),but serum E_2_ were significantly decreased (*P*<0.05). The levels of E_2_ of patients with blood stasis syndrome (BSS) were decreased further (*P*>0.05), and there was an increasing trend of serum sICAM-1 levels (*P*>0.05). There were negative significant correlations between serum E_2_ levels and TC, sICAM-1 levels in patient with coronary heart disease.

**Conclusion:**

The estrogen level of menopausal women with coronary heart disease is lower than healthy menopausal women. With the low estrogen levels, postmenopausal women tend to have high levels of blood lipids and sICAM-1, which elucidates that the estrogen could regulate lipids and attenuate inflammatory response to play a protective role on blood vessels.

## INTRODUCTION

With the improvement of living standards and aging population growth, the incidence of atherosclerosis keeps increasing year by year.^1^^,^^2^ The incidence of cardiovascular disease in middle-aged women is lower than middle-aged men, but the morbidity of postmenopausal women has gradually increased, and the incidence of 65 to 70 years old female is equal to male.^3^ Because estrogen can protect the vascular endothelial cells,^4^ inhibit the proliferation of vascular smooth muscle cells proliferation,^5^ reduce blood fat,^6^^,^^7^ and help prevent clotting.^8^ The decline of estrogen is one of the important reasons which leads to coronary heart disease in postmenopausal women. Blood stasis syndrome (BSS) is the important pathological procedure of occurrence and development of cardiovascular disease. In this study, we took postmenopausal women with coronary heart disease as research subjects and explored the relevance between blood stasis and estrogen levels to provide a new target and ideas for the prevention and treatment of cardiovascular disease in postmenopausal women.

## METHODS

***Reagents: ***Electrochemiluminescence assay kit of estrogen was from Roche Diagnostics GmbH, Germany (batch NO. 167224-01), and Enzyme-linked immune sorbent assay (ELISA) kit of soluble intercellular adhesion molecule 1(sICAM-1) was from R&D, USA (batch NO. 10-31-421).

***Diagnostic Standard: ***All the patients were diagnosed as having coronary heart disease (CHD) according to diagnostic criteria established by AHA and ACC (1999 version). The BSS cases had to conform to the 1986 revised diagnostic criteria.

***Inclusion Criteria:*** The selected patients were the postmenopausal women who were 45 to 75 years old, natural menopause more than one year, and on their first visit a doctor for CHD.

***Exclusion Criteria: ***Excluded were patients with serious infections, autoimmune diseases, severe ventricular dysfunction (EF <35%), grade III uncontrolled hypertension, severe vascular heart disease, diabetes, those with severe diseases of liver, kidney, hematopoietic system, primary nervous system or mental illness or cancer, women having medical history of major reproductive system disease. Especially the patients who had previous long-term use of lipid-lowering drugs, antiplatelet drugs for secondary prevention and therapy of coronary heart disease should be excluded. Those who were taking estrogen or had taken estrogen and were not in the washout period were also excluded.

***General Data and Grouping: ***In total, 43 patients with CHD were enrolled from Beijing Anzhen Hospital during April 2012 to January 2013, aged 45 to 75(64.01±6.60 years), who were divided into two groups: the BSS group (23 patients with BSS) and the NBBS group (20 patients with non-blood stasis syndrome). Meanwhile a normal control group consisted of 25 healthy postmenopausal women randomly selected from the health checking of Xiyuan Hospital, aged 45 to 75 (62.30±5.11 years), without any diseases in the heart, brain, liver, kidney, thyroid gland, without hypertension, hyperlipidemia and diabetes mellitus (this was made sure with physical examination, blood routine test, laboratory examination on biological indexes, B-ultrasonic examination and X-ray chest film and electrocardiogram).All the subjects enrolled in this study gave informed consent. This study was approved by the Hospital Ethics Committee.

***Data Collection and Indicator detection: ***The data included age, weight, height, smoking history, menopausal time, history of hypertension, diabetes mellitus, hyperlipidemia.All the patients were taken fasting venous blood for inspection at 8:00 the next morning after admission. The routine indicator was detected by Beijing Anzhen Hospital’s laboratory. The serum was separated for the testing of estradiol and sICAM-1 and stored at -80ºC. The estradiol was analyzed with electrochemiluminescence assay by Xiyuan Hospital’s laboratory and the level of sICAM-1 was analyzed with ELISA. Operation tests were all carried out according to the kit instructions strictly.

***Statistical Analysis: ***Measurement data which conformed the normal distribution were expressed as the mean±standard deviation (mean±SD), the one which didn’t conform the normal distribution were expressed as median (maximum, minimum). The non-normal distribution was converted into normal distribution by using Ln logarithmic conversion. Differences between two groups were tested using the two independent samples t-test. Differences among groups were tested using One-way ANOVA, followed by multiple comparisons by LSD test. The correlations between the indicators were analyzed by Pearson correlation analysis. All calculations were performed with SPSS version 17.0 software, and* P *values less than 0.05 were considered statistically significant.

## RESULTS

***Comparison of Baseline Data between Groups: ***There was no significant difference among the healthy control group (Control), the blood stasis syndrome group (BSS), and the non-blood stasis syndrome group (NBSS) in age, body mass index (BMI), menopausal time, and smoking history (*P*> 0.05).There was no significant difference between the BSS group and NBSS group as regards hypertension, dyslipidemia, type 2 diabetes(*P*> 0.05). (Table-I)

**Table-I:** Comparison of baseline data between groups (mean±SD or case)

**Group**	**n**	**Age** **(y)**	**BMI**	**The Menopausal Time(y)**	**Smoking** **(case)**	**Hypertension** **(case)**	Dyslipidemia** (case)**	**Type 2 Diabetes (case)**
Control	25	62.30±5.11	24.89±2.24	10.97±6.27	0	—	—	—
BSS	23	63.83±6.74	25.54±2.98	13.97±8.07	1	19	20	15
NBSS	20	64.19±6.45	24.93±3.01	11.72±5.99	1	17	20	13

***Comparison of E***_2_ ***, TC, TG, sICAM-1 between the control group and CHD group: ***Compared with the healthy control group, the levels of TG and TC in CHD group was significantly increased (*P*<0.01), the level of E_2_ in CHD group was significantly decreased (*P* <0.05), the level of sICAM-1 in CHD group tend to increase (*P*>0.05).(Table-II)

**Table-II:** Comparison of E_2_, TC, TG, sICAM-1 between the control group and CHD (mean±SD or median (max, min))

**Group**	**n**	**TG/(mmol/L)**	**TC/(mmol/L)**	**E**_2_ **(ng/L)**	**sICAM-1(ng/L)**
Control	25	1.04(0.64, 2.20)	4.09±0.12	56.18±17.11	306.93±20.05
CHD	43	1.68(0.44, 3.85)▲▲	4.83±0.16▲▲	39.37±11.63▲	321.68±23.58

▲ *P*<0.05,

▲▲ *P*<0.01, compared with the healthy control group.

***Comparison of E***_2_ ***, TC, TG, sICAM-1 between the BSS Group and NBSS Group: ***Compared with the healthy control group, the levels of TC and TG in BSS group and NBSS group were significantly increased (*P*<0.01 or* P*<0.05), but there was no difference between the two groups (*P*>0.05).

Compared with the healthy control group, the levels of E_2_ in BSS and NBSS group were significantly decreased (*P*<0.05), the level of E_2 _in BSS tend to be lower than the one in NBSS group (*P*>0.05).

Compared with the healthy control group, the level of sICAM-1 in BSS was significantly increased (*P*<0.05), but there was no significant difference between the healthy control group and the BSS group. There was no difference between the BSS group and the NBSS group (*P*>0.05). (Table-III)

**Table-III:** Comparison of E_2_, TC, TG, sICAM-1 between the BSS Group and NBSS Group (mean±SD or median (max, min)).

**Group**	**n**	**TG/** **（** **mmol/L** **）**	**TC/** **（** **mmol/L** **）**	**E**_2_ **(ng/L)**	**sICAM-1(ng/L)**
Control	25	1.04（0.64, 2.20）	4.09±0.12	56.18±17.11	306.93±20.05
BSS	23	1.68（1.10,3.85）▲▲	4.81±1.08 ▲	36.60±10.801 ▲	326.42±25.79 ▲
NBSS	20	1.57（0.44,3.75）▲▲	4.86±1.18 ▲	42.56±12.001 ▲	316.24±20.00

▲ *P*<0.05,

▲▲ *P*<0.01, compared with the healthy control group.

**Fig.1:**
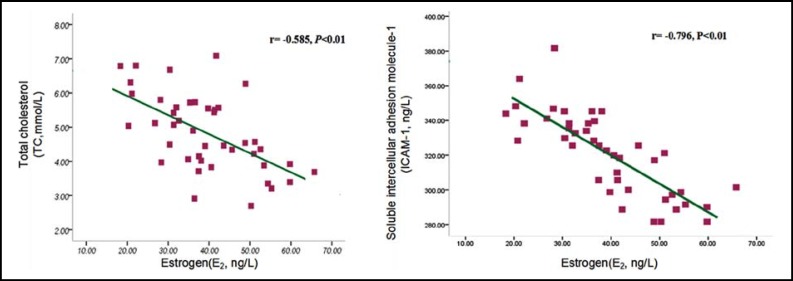
Correlations between Serum E_2 _Levels and TC, sICAM-1 Levels in Postmenopausal Women with Coronary Heart Disease.

**Fig.2:**
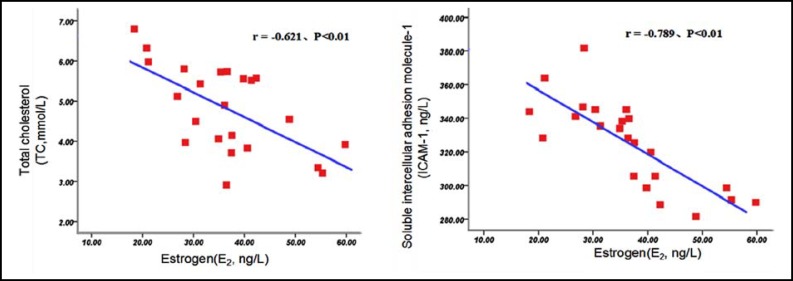
Correlations between Serum E_2 _Levels and TC, sICAM-1 Levels in BSS Group

**Fig.3:**
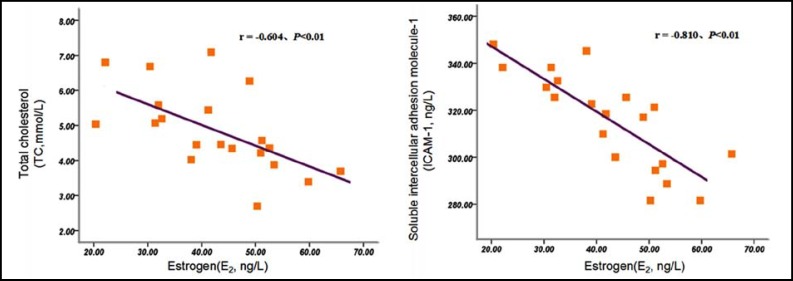
Correlations between Serum E_2 _Levels and TC, sICAM-1 Levels in NBSS Group

***Correlation Analysis: ***Pearson correlation analysis showed that there were negative correlations between serum E_2_ levels and TC, sICAM-1 levels in postmenopausal women with coronary heart disease (r = -0.585, r = -0.796, *P* <0.01). (Fig.1) Among them, there were negative correlations between serum E_2_ levels and TC, sICAM-1 levels in BSS group(r = -0.621, r = -0.789, *P *<0.01), and there were negative correlations between serum E_2_ levels and TC, sICAM-1 levels in NBSS group(r = -0.604, r = - 0.810, *P*<0.01). (Fig. 2 and 3) Spearman correlation analysis showed that there were no correlation between E_2_ and TG in postmenopausal women with coronary heart disease (r = -0.131, *P*> 0.05).

## DISCUSSION

Studies have showed that postmenopausal women with coronary heart disease can benefit from estrogen replacement therapy.^9^ In early menopause, estrogen replacement therapy or estrogen-progestin combination therapy on postmenopausal women with CHD could reduce the level of LDL-C and increase the level of HDL-C.^10^ This effect of estrogen may act directly on the vessel wall by combining with the estrogen receptor to affect the deposition of lipids in the vessel wall. Estrogen combines with the receptor on the liver cells, which activates the enzymes affecting the lipid metabolism, accelerates the clear of remnant chylomicrons, promotes the uptaken of remnant VLDL and LDL, facilitates bile acid secretion, removes cholesterol, increases apoA synthesis, and elevates plasma HDL-C.^11^

Compared with the healthy postmenopausal women, the level of E_2_ in postmenopausal women with coronary heart disease was decreased, but the levels of TC and TG were increased.^12^ The blood stasis syndrome is the main type of TCM types for CHD.^13^ Previous study had showed that compared with the healthy control group, the levels of TC, TG, and LDL in patients with CHD were all increased, and the HDL level was decreased; the lipid metabolism disorder in patients with phlegm turbidity syndrome was serious than the patients with BSS.^14^ The level of Lp(a) in CHD patients with BSS was significantly higher than the patients with non-blood stasis syndrome, which elucidated there might be relationship between the BSS and Lp(a).^15^ In all types of CHD, the level of TG in patients with BSS was higher than the patients with phlegm turbidity syndrome, but the level of HDL was lower than the latter.^16^ There are few studies to observe the relationship between E_2_ and lipid or inflammtory factors between the healthy menopausal women, menopausal women with blood stasis syndrome, and menopausal women with non-blood stasis syndrome.

In this study, the levels of serum TC and TG in postmenopausal women with CHD were significantly higher than in the healthy postmenopausal women, and there was a negative collection between E_2_ and TC. In addition, the study also investigated that the levels of E_2_ in CHD patients with BSS and patients with non-BSS were lower than the one in healthy women, but the levels of TC and TG were decreased; the level of E_2 _in patients with BSS were the lowest among all the groups. The plasma E_2_ in patients with BSS and patients with non-BSS were all negatively correlated with TC. This study further confirmed the effect of estrogen on lowering lipid, which prompted that the levels of lipids could be inferred according to the level of E_2_, but there is lack of research on the relationship between estrogen and severity of coronary heart disease, so people could not infer the severity of CHD on the basis of E_2_ level. 

Under the physiological state,^17^ vascular endothelial cells can maintain the tension and structure of the vascular, regulate blood clotting and fibrinolytic system, involve in inflammation and immune reaction, regulate the proliferation of vascular smooth muscle cell, regulate leukocyte and platelet adhesion on endothelial surface, regulate lipid oxidation and vascular permeability. The function of the vascular endothelial cells are affected, when the cells are damaged or activated, which lead to the vascular tone and vascular inflammatory changes, vulnerable plaque and thrombosis. Above is one of the mechanisms of the coronary blood stasis pathology.

Intercellular adhesion molecule-1 (ICAM-1) belongs to the immunoglobulin super family. A variety of cells, such as vascular endothelial cells, leukocytes, epithelial cells can express ICAM-1, and vascular endothelial cells have the strongest expression. It is the expression of the ICAM-1 in fingerprints and fibrous plaques of patients with AS that is the early pathological changes of AS and the potential mechanism of Plaque development, which is correlated with the severity of AS. Expressed ICAM-1 on the cell surface sheds into the blood to become sICAM-1. The amount of sICAM-1 is proportional to the number of cell surface molecules, and the concentration of sICAM-1 was indirectly reflecting the expression of ICAM-1 on endothelial cells and antigen-presenting cell surface. A large amount of ICAM-1 expresses on the surface of endothelial cells, and binds with ligand on the inflammatory cell surface, which helps the inflammatory cells attach to the endometrial cells to provide the conditions for further infiltration. The expression of ICAM-1 in the plaque of patients with CHD is significantly increased.^18^ Oral estrogen can reduce serum sICAM-1 to prevent the early process of atherosclerotic.^19^ A number of studies have confirmed that compared with the non-blood stasis syndrome group and healthy control group, the levels of hs-CRP, sICAM-1, sVCAM-1 in coronary blood stasis syndrome group are significantly increased.^20^^-^^24^

On the basis of the above, this study chose postmenopausal women with coronary heart disease as the research object and found that compared with the healthy postmenopausal women, the serum sICAM-1 tended to increase in postmenopausal women with coronary heart disease, and E_2_ and sICAM-1 levels were negatively correlated in CHD group. Further subgroup analysis found that serum sICAM-1 levels were incremental increased in the healthy control group, non-BSS group and BSS group, and sICAM-1 level in BSS group was significantly higher than the other two groups, which indicated that during the pathological process of atherosclerosis, patients with coronary blood stasis syndrome were subjected to more severe chronic inflammatory reactions than the patients with non-blood stasis syndrome, and E_2 _levels and sICAM-1 were negatively correlated. The study showed that estrogen may improve lesion of atherosclerosis by regulating endothelial function, reducing the adhesion between white blood cells, platelets and endothelial cells.

In conclusion, the level of E_2_ in postmenopausal women with CHD was lower than the level in the healthy postmenopausal women, and the CHD patients with BSS had a lower E_2_ level further, which could infer that the postmenopausal women who had a lower E_2_ level were susceptible to cardiovascular disease. When endothelial cells are damaged and activated, they increasingly secret adhesion molecules, which has close relationship with BSS. There are correlation between adhesion molecules and BSS. Estrogen could lower blood lipid and plasma sICAM-1 to regulate the vascular endothelial function and the adhesion between platelets, leukocytes and endothelial cell. However, the mechanism of vascular protective effects needs further study.

## AUTHORS CONTRIBUTION

Xin Liu conceived, designed and did statistical analysis and manuscript writing.

Chunyu Guo, did data collection and editing of the manuscript.

Xiaojuan Ma, Rui Tian, Ying Zhang, did data collection.

Huijun Yin, did review and final approval of manuscript.

Xin Liu takes the responsibility and is accountable for all aspects of the work in ensuring that questions related to the accuracy or integrity of any part of the work are appropriately investigated and resolved.

## Notes

***Note: ***Supported by National Natural Science Foundation of China (No. 8117358 4) and Beijing Natural Science Foundation (No.7122157).
